# Inequity in access to palliative care services worldwide and in Slovenia

**DOI:** 10.2478/raon-2026-0009

**Published:** 2026-02-04

**Authors:** Nena Golob, Maja Ebert Moltara

**Affiliations:** Department of Acute Palliative Care, Institute of Oncology Ljubljana, Ljubljana, Slovenia; Faculty of Medicine, University of Ljubljana, Ljubljana, Slovenia

**Keywords:** palliative care, inaccessibility, opioids, basic, specialized, Slovenia

## Abstract

**Background:**

Palliative care aims to enhance the quality of life of patients and their families facing progressive and incurable disease by addressing physical, psychological, social, and spiritual challenges. Despite being recognized as a human right, palliative care remains out of reach for most people worldwide, with only about 14% of those who need it receiving it. Global demand for palliative care is rising due to aging populations and the increasing burden of chronic diseases. While high-income countries focus on expanding access and inclusivity to this care, low-income countries face severe shortages in prevention, diagnostics and treatment of underlying diseases, which creates an urgent need for palliative care services. Cultural differences, a lack of trained professionals, limited opioid availability, and weak policy further deepen inequities.

**Conclusions:**

Historically rooted in religious and charitable care, modern palliative care emerged with Dame Cicely Saunders’ hospice movement, evolving into a medical specialty. Access varies widely – Europe has high integration in some countries but significant disparities in service distribution and opioid use. Africa, Latin America, and parts of Asia still lack widespread provision. In Slovenia, palliative care development began in the 1980s and has recently expanded to include some specialized palliative care services across the country. Despite this progress, palliative care in Slovenia remains underdeveloped due to limited coverage, regional disparities, workforce shortages, insufficient formal education, and an old and ineffective national policy. Opioid availability is slightly below the European average, and its use is declining, which raises concerns about further unmet needs.

## Introduction

Palliative care (PC) is a holistic approach that enhances the quality of life of patients and their families facing a progressive and incurable disease by addressing physical, psychological, social, and spiritual challenges. Interventions offer practical support and bereavement counselling, providing person-centered care with specific attention to individual needs and preferences. In accordance with the principle of equally respecting life and death, PC’s goal is to ensure a peaceful and dignified death, enabling patients to live as actively and fully as long as possible.^1^ The integration of PC into standard care also improves the quality of life for caregivers.^2^ Ultimately, PC services are intended to provide continuous support in a variety of settings to patients of all ages who are affected by a wide range of conditions.^1^

PC is recognized as a human right, yet only about 14% of people in need of PC receive it.^1,3^ Around 56.8 million people currently require PC services, and the global need for this care will continue to grow due to aging populations and the rising burden of noncommunicable diseases.^1,4,5^ Adequate national policies, programmes, resources, and training are urgently needed to improve access to PC services.^1,5^

## History of palliative care

PC has adapted to the needs of an evolving society: from its beginnings as houses dedicated to caring for ill and dying travellers to the first hospice-type facilities set up by the *Knights Hospitalier* and later by various religious organizations (*Irish Religious Sisters of Charity*), and finally to the establishment of the first modern hospice, *St. Christopher’s*.^6–8^ Dame Cicerly Saunders, largely credited for inspiring the modern hospice movement, worked with other distinguished professionals to establish a professional evolution of the field, which was recognized as a medical (sub)specialty in UK in 1987.^9^ Following the initial enthusiasm, critics from within and outside the specialty raised concerns about its limited outreach, the medicalization of death, and its role as a counterculture.^10–12^ Alongside with the partial inclusion of the hospice movement into National Health Service in the UK, pioneers of PC worked to promote their goals in many countries.^13^

PC has had to adapt to the new demands of a society with growing treatment possibilities. This is also reflected in how its definition and conception have evolved over time.^1,14^ Demands are further increasing due to geographic, cultural and societal challenges that are unique to the area where they are applied.^5,15^

In high-income countries, the emphasis of PC is on expanding access and ensuring the inclusion of all patients – regardless of their age, diagnosis, culture, race or minority status – through the provision of better access and patient-centred care. In contrast, many parts of the world still struggle due to limited resources for the prevention, diagnosis, and treatment of underlying diseases, which creates an urgent need for PC.^16–23^

## Inequity in global access and format

In low-income countries, disease (cancer) prevention, diagnostics and treatment are severely limited, making palliation the best option for most patients.^24^ Although PC plays a crucial role in improving the quality of life of patients everywhere, most of services are available and provided in high-income countries.^4,5^ Access in many other countries remains low.^5,25^ The reasons for this could lie in a lack of resources, limited awareness of the services’ existence, as well as the unavailability of PC services.^26–30^ Existing PC models are implemented mainly in high-income countries and are often incongruent with cultural issues in countries where they are applied.^31–34^ The task of providing “total care” may lead to services appearing radically different from country to country or even between communities.^35^ Issues among health care providers are important obstacles preventing the acquisition of PC.^26,36,37^ How PC is offered and received continues to be shaped by culture and ethnicity.^38–40^ The reluctance of patients and caregivers may also affect the delivery of PC.^41^

There are several models aimed at improving access to PC. The World Health Organization (WHO) *Global Atlas of Palliative Care* outlines various approaches to guide implementation across diverse health systems.^4^ In resource-constrained settings, programs may benefit from leveraging volunteers to expand accessibility.^35,42^ In contrast, specialist PC in high-income countries has been shown to improve patient and family satisfaction more effectively and to better address their needs compared to generalist PC.^43,44^ While access to specialist PC is often used as a benchmark for quality in high-income countries, in low-income countries, the mere provision of any form of health service is frequently considered a significant achievement.^45^ In these low-resource settings, PC is often focused on providing long-term support.^35^

A global study revealed that PC has been well integrated only into 20 health care systems worldwide. Nearly half of all countries lack a functional PC delivery system, and in many others, services reach only a small fraction of the population.^46^ In a report from 2008, 115 out of 234 countries had no PC services.^47^ Recently, notable progress has been achieved, particularly in Africa.^4^

Significant inequities exist in cancer pain management between low- and high-income countries. In low-income settings, most patients die in pain without access to adequate opioid pain treatment, whereas this rarely occurs in high-income countries. Most of the world’s morphine consumption takes place in high-income regions. The use of opioids is closely linked to the availability of PC services, as the presence of a functional system is essential to ensure that even when medications are available, they are appropriately prescribed and delivered.^5,48–50^

In Africa, patients with chronic diseases face a high prevalence of pain and psychosocial distress, yet PC services remain largely unavailable. Currently, 21 out of 47 countries have no PC provision, while only about 22 countries have made some progress towards integrating PC into their health systems.^4,46^ Access to essential medications remains a critical barrier, with oral morphine still largely unavailable across the continent.^4,51,52^

In recent years, PC services have expanded in Latin America, and all countries on the continent now offer some form of provision. However, comprehensive national policies and strategic plans remain rare. The development of services is highly uneven, with nearly half located in Argentina and Chile – countries that together represent only about 10% of the continent’s population. These services are typically concentrated in major urban centres and are often linked primarily to cancer care. Despite ongoing efforts, access to and availability of PC remain limited for most of the population across Latin America.^50^

In recent years, the state of Kerala (India) has made remarkable progress in PC integration, largely driven by a strong network of communitybased initiatives. Community volunteers play a crucial role there, serving as a vital link between patients and healthcare providers. They provide an estimated coverage of more than 70% of longterm care in the region, compared with a national average of 1–2% (35,42,53,54).^35,42,53,54^ The high rate of morphine use in Kerala is a direct reflection of its well-developed PC infrastructure.^53^

Although some studies report poor awareness of PC services among adults in the USA, primary care providers play an integral role in providing generalist PC, while specialist PC provides an extra layer of support.^27,29^ The relationship between generalist and specialist PC is expected to deepen over time.^27,55^ Among adult Americans who experienced a period of dependency before death, 40% received care within hospice programs.^46^

While, according to reports, PC is approaching integration into the mainstream health care system in most European countries, there is a striking variation in the levels achieved in each country and between countries.^5,46^ A recent European study identified 7,119 specialist PC services and 524 paediatric services across Europe. PC is officially recognised as a medical specialty in 21 out of 52 countries, with mandatory training in medical and nursing schools in 19 and 21 countries, respectively. However, only 13 countries have a dedicated PC unit at the Ministry of Health, and just nine have a national PC law. PC policies are included in primary care services in 76% of countries, and 33 out of 52 countries host annual national PC conferences. Research activity is strong in 19 out of 52 countries. Advance care planning policies exist in 16 out of 52 countries, with eight allowing living wills and surrogate decisionmakers. Opioid use across Europe varies greatly, from fewer than 500 to more than 5,000 doses per million people per day, with an average of around 5,000 daily doses.^5^

## Palliative care in Slovenia

**FIGURE 1.**
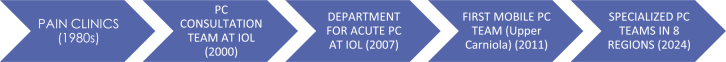
Palliative care evolution in Slovenia. IOL = Institute of Oncology Ljubljana; PC = palliative care

PC in Slovenia began in the 1980s with the establishment of hospital-based pain clinics (Figure 1). In 1996, the Slovenian Hospice Society was founded to support terminally ill patients and their families. The first formal PC consultation team was launched at the Institute of Oncology Ljubljana (OIL) in 2000, concurrently with the gradual expansion of PC activities into nursing homes, health centres, and other care settings (Figure 1).^56,57^

In 2006, the OIL published its first internal guidelines for the treatment of patients with advanced cancer, followed by the opening of the Department for Acute PC in 2007 (Figure 1).^56–58^ The adoption of the National Program for PC in 2010 provided a framework for structured development across the country, though regional progress has remained uneven. The program introduced the concept of basic and specialized PC (SPC) services.^56^

In 2011, Slovenia’s first mobile PC team (MPCT) was established in the Upper Carniola (Figure 1).^56,57^ This was followed by the launch of the first PC counselling service in 2012 and an early PC outpatient clinic at the IOL in 2013. The Slovenian Association for Palliative and Hospice Care, founded in 2011, has been instrumental in education and advocacy. It became a full member of the European Association for PC (EAPC) in 2014. In the same year, the Institute for PC at Medical Faculty of the University of Maribor was established to support research in the field.^56^

A major milestone was reached in 2021 when five hospitals signed the program for MPCT with the Health Insurance Institute of Slovenia. This was followed by the Ministry of Health beginning to implement a European-funded project focused on MPCT (“*Strengthening Mobile Teams*,” 2022–2026) (Figure 1). By the project’s conclusion in 2026, 14 MPCTs are expected to be funded. Simultaneously, additional efforts have been directed towards strengthening the entire network of services such as acute PC departments, outpatient clinics, and telephone support services across the country (Figure 1).^56,57,59,60^

PC in Slovenia is delivered by all healthcare professionals and the community (caregivers, non-governmental organisations). According to the National PC Programme, PC is regionally organized in Slovenia at the basic and specialized level.^59,61,62^ Basic PC is carried out at the primary level by general practitioners and district nurses, and at the secondary level in hospitals by specialists from any clinical fields in collaboration with the hospital nursing staff.^56,58,59,61,63,64^ SPC is intended for patients with complex and difficult-to-manage symptoms and specific needs. Only a small proportion of patients and caregivers (20%) require SPC, which is provided in secondary and tertiary health centers, and by MPCT.

SPC is provided by multidisciplinary teams comprised of professionals from various medical fields (e.g. clinical medicine specialists, nursing staff, pain management specialists, nutritional support professionals, clinical pharmacists, clinical psychologists and social workers) who have acquired additional education in PC. The core PC team consists of a physician and a nurse, but it is often expanded into a broader team that includes a social worker, physiotherapist, psychologist, and other professionals to better meet patient’s needs.^56,58,59,61,63,64^ A crucial member of the multidisciplinary SPC team is the PC coordinator, who oversees the services and offers telephone support for patients at home.^56,61,63,64^ In Slovenia, the indications for enrolment to SPC services include specific and complex physical, psychosocial, and spiritual needs of the patient or their caregivers. Specialized support in PC in addition to the care at the primary level (by the general practitioner and community nurse), is needed due to anticipated severe complications.^56,65,67^

As of 2024, there are SPC teams in eight statistical Slovenian regions (Central Slovenia, Upper Carniola, Drava, Savinja, Mura, Coastal-Karst, Gorizia, and Southeast Slovenia) offering different type of support. In the Central Slovenia, there are four SPC teams: for cancer patients at the OIL; for patients with hematologic conditions at the Department of Hematology of the University Medical Centre Ljubljana (UKC LJ); for children at the Pediatrics Clinic of the UKC LJ; and for patients with amyotrophic lateral sclerosis at the Neurological Clinic of the UKC LJ. In the Drava region, two distinctive SPC teams are operating, one driven by the University Medical Centre Maribor and one by the Ptuj General hospital.^58.59^ SPC teams perform home visits, consultation services, outpatient clinics and phone support. An updated list of SPC teams and the service they offer with contacts is available on www.paliativnaoskrba.si.

The only SPC hospital department that has been operating continuously since its establishment in 2007 is the Acute PC Department at the OIL.^58^ In the past, two other similar departments operated (in the hospitals of Nova Gorica and Slovenj Gradec), but they were closed during the COVID-19 pandemic. The department in the Slovenj Gradec hospital has recently reopened and nowadays offers an inpatient clinic in addition to phone support, consultation, and outpatient services.^56,58^

In the SPC Outpatient Clinic, patients with incurable and advanced disease who are still in a stable phase and suitable for outpatient referral with a waiting period are referred based on a triage referral. Patients who are bed bound and not suitable for an outpatient clinic visit are managed in their home environment by the MPCT.^58^

A referral for SPC services can be made by a physician of any specialty – mostly oncologists, general practitioners, and emergency medicine physicians – or by others involved in the patient’s care who require assistance in managing complex symptoms and specific needs.^58^

Based on the National PC Programme, one SPC mobile team should assist 100,000–150,000 inhabitants.^59^ Currently, due to high demand and insufficient resources, first home visits are often delayed. Telephone availability is seldom 24/7, and there is a waiting list for outpatient and counseling services.^58,67^

Currently, there is only one inpatient hospice in the country.^58^

Although there have been major improvements over the years, new problems have arisen.^5,25,58,62,68^ Due to a growing shortage of general practitioners, basic PC in Slovenia is often poorly accessible for patients. Consequently, SPC team, although already short-staffed, are also assuming the care of patients with basic needs.^58,68^

According to the EAPC Atlas launched in June 2025, PC in Slovenia is underdeveloped, providing only isolated PC provision. At the time of the report (October 2024–March 2025), there were 10 PC teams nationwide for adults, which translated to 0.47 services per 100,000 people. This was well below the European median of 0.96 services per 100,000 people, with limited access to hospital care, hospice and home-based PC. There was only one pediatric team nationwide at the UKC LJ with additional pediatric PC teams are in the process of being set up at regional hospitals. In Europe, there were 524 SPC teams in total, present in 41 out of 52 countries.^5^

Education and training in PC are minimal: by the end of 2024, no medical schools (0/2) offered obligatory formal PC training, and only one nursing school did (1 out of 9). There are no PC professors in the country and no advanced PC educational programmes, such as (sub)specialisation for physicians. The 40-hour training for residents is recommended for some specialties.^5^ The Slovenian Association for Palliative and Hospice Care holds a 60-hour PC course for professionals and also organizes a national conference every second year.^5^

There are several groups promoting the rights of patients in need of PC, their caregivers, and disease survivors in Slovenia, but despite this, advanced directives are not part of clinical everyday practice.^5^

According to EAPC Atlas, while there are some cancer-related and paediatric PC initiatives, there is still no national PC law or renewed official PC legalisation.^5^

Opioid availability in Slovenia is slightly below the European average. Access to essential PC and pain medications remains strong across both urban and rural settings. There is a marginal disparity, with rural areas having slightly lower access to immediate-release oral morphine compared to urban areas. Nonetheless, the overall availability of various opioid types and formulations at the primary level remains high.^5^

However, a national database study revealed that the prevalence of opioid recipients has recently decreased in Slovenia, and the overall consumption is lower than the European average.^5.69^ The low prevalence rises concerns about the extent to which PC needs are being met in the country.

## Conclusions

Demographic changes, evolving survival trends and a widening gap in resources are just some of the factors that will require further adaptation of PC provision worldwide and in Slovenia.

Although there have been questions whether PC is well-provided and organized, and whether specialist PC is justified, PC does indeed improve the quality of life for patients with incurable and progressive diseases, enabling them to live as actively and fully as possible until death.

Inequities in PC are a result of geographical, cultural and societal factors. This heterogeneity needs to be addressed by properly providing adequate modalities and approaches of care in different settings. In low-resource settings, community participation can be a tool for empowerment, serving as a link between the patient and healthcare providers. Volunteers could be actively involved in evaluating, monitoring, and modifying existing programmes. In high-income countries, specialist PC should be enhanced by providing better access to all patients, regardless of their age, diagnosis, culture, race, or minority status.

In Slovenia, a further expansion of SPC services is needed to provide adequate and equal care for patients all over the country. Basic PC must be ensured by all levels of healthcare. Educational programmes for professionals could therefore facilitate PC provision across the country, as this is our greatest responsibility. The main goal of national policies and all healthcare providers is to ensure person-centred care and to identify and overcome barriers that slow down the development and integration of these services.
